# The Efficacy of Phonological Processing Treatment on Stuttering Severity in Persian Pre-School Children

**Published:** 2019

**Authors:** Neda TAHMASEBI, Mohadeseh RASTI BORUJENI, Majid SOLTANI, Mahmoud LATIFI, Negin MORADI

**Affiliations:** 1 Musculoskeletal Rehabilitation Research Center, Ahvaz Jundishapur University of Medical Sciences, Ahvaz, Iran; 2 Diabetes Research Center, Health Research Institute, Ahvaz Jundishapur University of Medical Sciences, Ahvaz, Iran

**Keywords:** Stuttering, Treatment, Phonological processing, Stuttering severity

## Abstract

**Objectives:**

Correct phonological encoding is crucial to fluent speech production. Phonological working memory and phonological awareness are important phonological processes that affect phonological encoding. The purpose of this study was to investigate the effect of phonological processing on stuttering severity of Persian pre-school children.

**Materials & Methods:**

Six children were targeted in this study in Ahvaz City, southern Iran in 2018, with Quasi-experimental design (Before and after clinical trial). These children participated in a treatment protocol, scheduled in 13-sessions. The treatment protocol of the phonological processing included nonword repetition in the phonological working memory and phonological awareness therapy. Overall, 30 nonwords were taken to examine the phonological working memory. The Persian test of language development was taken to examine phonological awareness. Stuttering severity measurements were performed with pre- and post-treatment. The severity rating was instructed to the parents based on Guitar protocol. They were asked to keep score every day until the end of the treatment sessions, and they reported the score to the therapist.

**Results:**

The stuttering severity score in pre and post-treatment was significant (*P*=0.027), and in the follow-up, phase was not significant (*P*=0.236); stuttering severity was reduced in children who stutter. Moreover, the severity rating score reported by parents during treatment was significant (*P*= 0.0001). This showed a reduction in stuttering severity.

**Conclusion:**

The poor performance of phonological awareness and phonological working memory in phonological processing affect stuttering severity. Treatment of sub-systems of phonological processing can have an important role in reducing stuttering severity and increasing speech fluency.

## Introduction

Stuttering is generally known as a multi-factor phenomenon (1). Given the complexity of stuttering, many assumptions have been proposed regarding its origins (2). One of the most comprehensive psycholinguistic theories on the cause of stuttering is the Covert Repair Hypothesis (3). Based on the Levelt model, this hypothesis suggests the process of word production and seeks to investigate the causes of stuttering, including repetition of sound, prolongation, and blocking during the fluent speech (4). According to the Levelt model and covert repair hypothesis, before phonic production, correct phonological encoding is important for fluent speech production (5-9). 

Phonological processing, as an umbrella term, includes various skills, which have many effects on phonological encoding (10). Phonological working memory and phonological awareness, which are part of phonological processing, are factors affecting phonological encoding (1, 8, 11). Working memory is a neuro-cognitive system that includes the central executive, a phonological loop, a visuospatial sketchpad, and an episodic buffer (2, 9, 12, 13). Phonological loop plays an important role in phoneme encoding and correct phoneme retrieval in the working memory (1, 5). Another factor in phonological processing is having phonological awareness skills (8). Phonological awareness skills refer to an individual's ability to analyze, recognize, discriminate, and manipulate phonemes in speech, not considering the word size and meaning (8). 

Studies have focused on the factors influencing phonological encoding and, by examining the results; phonological processing can be affected by various language and speech disorders, which cause phonological encoding disorders (14-16). One of the speech disorders is stuttering (5, 8, 12, 13, 17). Phonological awareness studies were conducted on children who stutter (CWS). Phonological encoding ability in CWS was examined. There are differences in phonological awareness ability of the two groups of children who do and do not stutter (8). Studies in the field of phonological working memory have investigated the phonological loop, which is part of the active phoneme memory, in people who stutter (PWS). These studies used a nonword repetition task to examine this part of the working memory. Phonological working memory performance in PWS showed weaker than people who do not stutter (PWNS) (1, 2, 12, 18-20). 

 The present study was conducted to investigate the effect of phonological processing treatment on the reduction of stuttering severity of pre-school children. The aim of this study was to evaluate and cure the non-verbal dimensions in order to enhance speech fluency of children who stutter. 

## Materials & Methods

This study with Quasi-experimental design (Before and after clinical trial) targeted six children who stutter (3 girls and 3 boys) with an average age of 6 yr and 4 months. The participants of this study were selected from among the CWS referrers to Speech Therapy Clinics of Ahvaz Jundishapur University of Medical Sciences in 2018. 

The study was approved by the ethics Committee of Ahvaz Jundishapur University (NO. IR.AJUMS.REC.1395.765), and informed consent was taken from the parents of children who stutter. Before the entrance of the children, their parents signed the letter of consent for the study. 

Furthermore, the children along with their parents would be able to opt out of continuing to collaborate with at any time. The children's information was also completely confidential.

The inclusion criteria for the study were a weakness in the phonological awareness skills and phonological working memory. The parents of CWS reported no history of cognitive, mental and sensory, lingual, hearing, production, and short-term memory defects. Moreover, none of the participants had received treatment before participating in the study. Other inclusion criteria were: at least 6 months of stuttering and confirmation of stuttering by a speech and language pathologist, observing at least 3 dysfluencies per 100 spontaneous speech, and confirmation of stuttering by a parent or the child’s instructor. 

To assess the phonological awareness skills of the subjects, Soleymani-Dastjardi Persian version of phonological awareness scale was used (21). The nonword repetition task consisted of 30 nonwords was taken to examine the phonological working memory (5). Persian test of language development (TOLD) (22), auditory discrimination subtest (22), phonetic test (23), digital span test, Wechsler Intelligence test (24), and the Persian version of SSI4 (25, 26) were other tests used to evaluate the participants in this study. The reliability of phonological awareness skills test was calculated through two methods of test-retest and Cronbach's alpha of 0.990 and 0.982 (21). The nonword repetition task, 30 nonwords carried out in a study were done on children 100 nonwords made by changing one or two consonants of Persian words so that they do not convey a specific meaning. The phonetic structures of these nonwords had also been adapted to the common Persian phonetic pattern (CV, CVC, and CVCC). Nonwords were given to 15 normal children aged 5 to 8 yr. Among them, 40 nonwords repeated correctly (just at the first attempt) by 85% of children in a ‘without time pressure’ condition were selected for use in the experiment. Because scoring has not yet been determined for this test, considering the first quarter of the total score of 30, children who scored below 10 were included in the study because they were considered weak in this ability (5). Persian test of language development showed validity over 0.9 (22). This auditory discrimination subtest was calculated with the differentiation power of 0.80 in the Persian language development test (22). Phonetic test was calculated with a correlation span test was calculated by methods of split-half and test-retest 0.71 (24). The reliability of Stuttering Severity Instrument was calculated through two methods of inter and intrajudge higher than 0.90 (26).

Before starting intervention sessions, one week was allocated to evaluate the CWSs. One week before starting treatment, the Persian version of SSI4 was performed for 3 sessions in order to determine the children’s stuttering severity. In order to assess the level of phonological awareness of the children, a standardized phonological awareness test was used (21). Prior to the treatment, the severity rating was trained to the parents based on the Guitar protocol, and the agreement of parents and the speech-language pathologist on the severity of stuttering was investigated. The parents were asked to take a score every day until the end of the stuttering sessions, and report the score to the therapist, with a score of zero (no stuttering) to 10 (highest stuttering) (27). 

Subjects were treated with 13 sessions of 45 min. The intervention was performed in two parts in each session.

**Treatment of phonological working memory**

In each session, first, the phonological working memory underwent treatment. Overall, 400 nonwords were presented to 16 subjects in 16 packages of 25 in 13 sessions. The nonwords were designed easy to hard, in the first three sessions because of the simplicity of nonwords, 2 nonword packages were provided in each session, and from the fourth session onward, in each session, a nonword package was provided to the subjects. Nonword packs were made based on the factors influencing non-word repetition, including non-word length, production complexity, and lack of vocabulary similarity and its content validity was confirmed by a linguist. Nonwords were created by changing one or two consonants of a Persian word to not transfer any particular meaning. The phonetic structures of the nonwords were adapted to the common Persian dialect pattern (CV, CVC, and CVCC) (28, 29).

The subjects were first instructed that a number of meaningless words would be expressed to them, and they should carefully listen to the meaningless words and repeat them immediately after the meaningless word was uttered. In order to ensure that the subject fully understood the steps involved in doing therapy tasks, 5 nonwords were stated as training items. After the training phase, nonword packets were expressed by the therapist and the subject repeated each nonword after hearing it. During each session, each packet was repeated 5 times, and the subject was given the opportunity to hear each nonword 5 times and try to repeat it more accurately. In each session, a new nonword packet was provided to the subject and in the event of failure in that package, an equivalent packet was presented for the next session (28, 29). 

**Treatment of phonological awareness**

The second part of the treatment sessions included phonological awareness therapy. Seven phonological awareness subtests were designed according to the age range and were given as 13 tasks to the subjects in 13 sessions. Overall, 300 illustrations of picture books of consonants and preschool books were collected by the researcher. These tasks were entirely based on the phonological awareness test (three components of syllable knowledge, intrasyllabic unit awareness, and phoneme awareness), subtests of fragmentation, concurrency detection, rhyming detection, phonemic combination, identifying words with the same initial phonemes, identifying words with the same final phoneme and naming and deletion of the end phoneme (21). In order to coordinate the treatment part of the phonological working memory with this therapeutic area during 13 sessions, two sessions were devoted for each phoneme awareness treatment part.

After the treatment, the Persian version of SSI4 was used to determine stuttering severity, nonword repetition task, and the number of correct nonword repetition in phonological working memory session Phonological awareness was used to compare the pre- and post-treatment test scores. The CWS phonological process treatment protocol is shown in Table 1.

**Table 1:** The Phonological Process Protocol for treatment of CWS

Treatment Protocol		
Before treatment	Pre-test	Nonword repetition task, The Persian version of SSI4, Phonological awareness test 16 packages of 25 nonwords:
	13 sessions of treatment in two parts	From the first to the third session, repeat 2 packages of nonwords, and from the fourth session onward repeat one package of nonwords until the end of each session
Treatment		14 educational packages from each sub-test two educational packages: In the first session, two packages of the first sub-test were used, while in the next 12 sessions, a therapeutic package of 6 remaining subtests were utilized.
Post-treatment	Post-test	Nonword repetition task, the Persian version of SSI4, Phonological awareness test
Follow-up		One week after the treatment

Statistical analysis

**SPSS software (ver. 21** **(Chicago, IL, USA)** **was used for statistical evaluation. The criterion for statistical significance was defined as** ***P*** **-value≤0.05. The Kolmogorov-Smirnov test was used to determine the normal distribution of variables. To compare the data the Nonparametric Wilcoxon test was used.**

## Results

**Figure 1:**
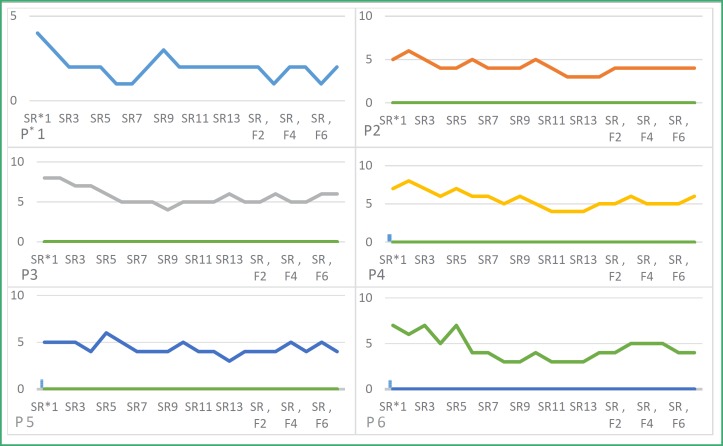
Changes in the rate of stuttering severity by parents in treatment and follow-up sessions

**The summary of the subjects' biography is presented in** Table 2 **and the comparison of the stuttering severity pre- and post-treatment are shown in** Figure 1 **. Drawing upon the results of SSI4 at the end of the intervention, and comparing the stuttering severity pre- and post-treatment, the reduction in stuttering severity was observed in all subjects.**

**Table 2:** Participants’ description

Participant	Gender	Age(yr)	Stuttering severity	Stuttering severity score pre-treatment	Stuttering severity score post-treatment
1.	Male	6.4	Mild	13	10
2	Female	6.4	Average	22	18
3	Male	6.9	Severe	28	24
4	Female	6.8	Average	25	22
5	Male	6	Mild	16	11
6	Female	6	Average	20	18

The Wilcoxcon test was used for statistical analysis of stuttering severity pre- and post-intervention. Table 3 shows the data before and after the treatment. The results of statistical analysis (*P*<0.05) showed a significant difference. Table 3 shows the mean, minimum, maximum, median, standard deviation and *P*-value resulting from the nonword repetition in the section of the phonological working memory and the 7 sub-tests of fragmentation, convergence, rhyme, composition, identification of the words with the same phoneme initials, identification of words with the same ending phoneme and naming and deletion in the phonological awareness section before and after the treatment.

**Table 3:** Scores obtained by subjects in the pre- and post-treatment

	Pre-treatment				Post-treatment				
	Minimum	Maximum	Mean±sd	Median	Minimum	Maximum	Mean±sd	Median	*P*-value
Number of correct repetition of nonwords	12	15	1.1±13.1	13	23	29	2.2±25.5	25	0.027
Fragmentation score	5	9	1.4±7.1	6.50	9	10	0.5±9.6	9.50	0.027
Rhyme score	3	6	1.2±5.3	6	7	9	0.8±8.3	8.50	0.024
Convergence score	4	5	0.4±4.8	5	7	10	1.03±8.3	8	0.027
Combination score	3	5	0.8±4.3	4.50	6	9	1.1±6.8	6	0.026
Identification of the words with the same initial phoneme	0	4	1.5±2.5	3.50	5	8	1.2±7.3	7.50	0.026
Identification of the words with the same final phoneme	2	6	1.6±3.8	4.50	6	9	1.1±7.8	7.50	0.026
The naming and deletion of the final phoneme	0	6	1.9±3.6	4	4	8	1.3±6.3	6	0.027
Stuttering severity	13	28	5.5±20.6	21	10	24	5.6±17.1	18	0.027

**Participant 1:** The mild stuttering severity before the treatment was reduced from score 13 to score 10 after the treatment. In addition to reducing the severity score, this participant was positioned in the very mild level in the rating table. Among the participants, only participants 1 and 5 had mild stuttering. Due to a lower stuttering score at the end of treatment, participant 1 showed better performance in phonological working memory and nonword repetition task. Moreover, among all participants, he obtained the highest score for the correct nonword repetition. In phonological awareness in all of the given subtests, he showed better performance after treatment, with the highest improvement in the section identification of the words with the same initial phoneme. He scored the highest in the naming and deletion of the final phoneme section among all the participants. Severity rating reports for the scores by the parents were reduced from 4 to 2. 

**Participant 2:** The average stuttering severity before the treatment was reduced from 22 to 18 after the treatment. In all subtests, recovery and improved score were observed. In the phonological awareness section, the major change was observed in the identification of the words with the same final phoneme. Severity rating reports for the scores by the parents were reduced from 5 to 3. 

**Participant 3:** The severe stuttering severity pre-treatment before the treatment was reduced from 28 to 24 after the treatment. In addition to reducing the severity score, this participant ranked average in the leveling table. For this participant, recovery was observed in all of the studied areas, and in the phonological awareness section, the most observed changes were detected in the identification of the words with the same initial phoneme. Severity rating reports for the scores by the parents were reduced from 8 to 5. 

**Participant 4:** The average stuttering severity before the treatment was reduced from 25 to 22 after the treatment. In all subtests, recovery was observed and the score was increased. In the phonological awareness section, the most significant change was observed in the identification of the words with the same initial phoneme. Severity rating scores by the parents were reduced from 5 to 3. 

**Participant 5:** The average stuttering severity before the treatment was reduced from 20 to 18 after the treatment. In all the subtests, recovery was observed and the score increased. In the phonological awareness section, the most significant change was observed in the identification of the words with the same initial phoneme. Severity rating scores by the parents were reduced from 5 to 3. 

**Participant 6:** The mild stuttering severity before the treatment was reduced from score 16 to score 11 after the treatment. In all the sub-tests, recovery was observed and the score increased. Like other participants, in the phonological awareness section, the most significant change was observed in the identification of the words with the same initial phoneme. Severity rating scores by the parents were reduced from 5 to 3. 

**Severity rating**

Figure 1 shows the severity rating of each subject from the beginning to the follow up scored by parents in each session.

SR*: Severity rating, SR, F*: Severity rating in follow up, P*: Participant

Friedman test (repeated measures) was used to analyze the severity rating provided by the parents. The result of the severity rating score of the subjects (*P*=0.0001) is significant.

**Follow-up**

 In order to evaluate the effect of treatment on stuttering severity of CWS, severity rating was recorded each day by the parents one week after treatment. Statistical analysis in the follow-up phase was done using the Friedman test, and the results were not significant (*P*=0.236). Therefore, no difference in treatment period and treatment stability was observed.

## Discussion

The aim of this study was to investigate the effect of phonological processing treatment on stuttering severity of Persian pre-school children and to assess their pre- and post-treatment scores. In general, the outcomes of this study include the scores obtained in phonological working memory, phonological awareness, and stuttering severity.

In short, the process of nonword production occurs in the phonological loop without the need for prior vocabulary knowledge. As selecting target phonemes from other phonemes during the production of a word in the phonological loop of PWSs takes time, the probability of an error in selecting a correct phoneme is increased (4, 5). In this study, phonological working memory in CWS was treated using a nonword repetition task. Measurement outcomes showed an increase in the mean of the nonword repetition task post-treatment. With respect to the main objective of the study, by increasing the correct response to the nonword repetition task, the effect of improving the phonological working memory on reduction of stuttering severity was observed. Therefore, our results showed that the number of correct responses to the nonword repetition task increased by reducing the probability of error incorrect phoneme selection and generally improving the phonological cycle. The results of this study were in line with studies on the weakness in the phonological cycle of children who stutter (1, 12, 20). PWS were less accurate in repeating nonwords than PWNS, and phonological working memory may contribute to the creation or maintenance of speech fluency in PWS (1). School-age CWS had a higher number of mistakes compared to those children who do not stutter (CWNS), the phonological working memory in CWS is weaker than CWNS (20). In another study, the reported results of phonological working memory on CWS showed a higher mean error, but there was no statistically significant difference between the two groups, which could be due to the low sample rate of the research. However, by observing the difference in the mean nonword repetition error in CWS and CWNS, there is a degree of delay in phonological working memory ability of CWS (especially a phonological encoding defect in word production) (9). This part of the present study is consistent with the weakness in the function of phonological working memory argued in previous studies.

The results of a similar therapeutic study performed using nonword repetition task in CWS showed that by decreasing the number of phonological errors and the reduction of corrections during speech production, phonological working memory, in the phonological encoding function is improved. Finally, dysfluency reduction in these children was reported. In addition to focusing on the nonword repetition task to improve phonological working memory, the study used a motivational plan to enhance participants' learning. Therefore, as mental and psychological factors have a very significant effect on the function of CWS, the reduction in the stuttering severity observed in the participants in the study was completely due to the improvement of phonological working memory (28). 

In the phonological awareness part, this study compared pre- and post-treatment scores of 7 sub-sets: fragmentation, identification of convergence recognition, rhyme, phonological composition, identification of words with the same initial phonemes, identification of the words with the same ending phonemes, and naming and deleting the final phoneme. Outcome measurement in this section showed an increase in the post-treatment scores was in all sections. An interesting point in measuring the outcomes was the greatest change and improvement in the subtests of identification of the words with the same initial phoneme and identification of the words with the same final phoneme in all subjects. The purpose of these two subtests was to detect the same sound at the beginning or end of the intended words in the test, by improving phonological processing in both phonological awareness and phonological working memory, the correct phoneme retrieval and recognition in the subjects are improved. This confirms the covert repair hypothesis in relation to the weakness of PWS in recognizing and recovering the correct phoneme from rival phonemes. The treatment results of a study on CWS aged 4-6 yr showed that these children had a significantly lower performance than CWNS in phonological awareness and suggested that CWS may show linguistic differences in different aspects of phonological encoding of linguistic programming (8). By comparing phonological awareness in CWS and CWNS, another study reported stuttering as an effective factor in reducing the score in the phonological awareness test (30). Outcome measurement of this study, in addition to confirming the weakness in phonological awareness of CWS, showed a post-treatment decrease in stuttering severity of the subjects. Therefore, this part of phonological processing, along with the treatment of phonological working memory, can be effective in reducing stuttering severity. Contrary to the results of this study on reduction of stuttering severity, in another study, with a weakness in the phonological awareness of CWS compared to CWNS, there was no significant relationship between phonological awareness and dysfunction in these children, which could be due to small sample size. However, special attention should be paid to the potentials of phonological awareness in evaluating and treating CWS (19).

The important aspect of this study was the comparison of the stuttering severity of CWS pre- and post-phonological treatment. Stuttering severity in SSI4 ranges from very mild to very severe, and each type of stuttering severity has a specific numerical range. By comparing the mean stuttering severity score before and after the intervention, a decrease in the mean stuttering severity score was observed in all subjects. Moreover, by doing statistical analysis on the severity rating by parents, the stuttering severity score in all subjects decreased. 

 Stable changes in stuttering severity were observed by comparing the average severity rating determined by the parent during the treatment and the weekly follow-up of the sustained effect of the treatment. However, given that the follow-up period of children was considered one week after the treatment, the score could be considered as a suitable measure for the assessment of the therapeutic sustainability. This treatment was performed as an experimental and indirect treatment of the stuttering severity of CWS. Therefore, a shorter follow-up period was considered to allow parents to go to treatment centers more quickly to continue the stuttering treatment. 

The limitations of this study include the number of participants, the duration of treatment, and the short follow-up period after the treatment, therefore, another intervention program simultaneously examines these two phonological processing components, i.e. phonological awareness and phonological working memory, on a larger sample of children with stuttering problems with varying severity degrees in separate groups and a longer treatment period, as well as a longer follow-up period. 

This research will help to plan the future treatment interferences in the treatment of PWS. It will also be important to focus on these two areas in order to expedite the treatment of children and prevent language problems that they will be encounter during school times.

**In conclusion, **the treatment of phonological processing on CWS may have an effect on decreasing the stuttering severity. The treatment of phonological processes, as an indirect treatment, can be included in therapy sessions, for example, along with Lidcombe's program. Due to language problems in the field of phonological awareness and phonological working memory, the treatment of this disorder, in addition to reducing the stuttering severity of these children, can also prevent their linguistic problems at school. However, despite the fact that all participants showed a decrease in stuttering, taking into account the samples size of the study, the effect of treatment is yet hard to be considered as certain. 
